# A wearable adaptive penile rigidity monitoring system for assessment of erectile dysfunction

**DOI:** 10.1038/s41378-024-00721-5

**Published:** 2024-09-20

**Authors:** Xiangyang Wang, Ruojiang Wang, Yuyang Zhang, You Wu, Xu Wu, Zihao Luo, Yu Chang, Xiansheng Zhang, Tingrui Pan

**Affiliations:** 1 School of Biomedical Engineering, Division of Life Sciences and Medicine, University of Science and Technology of China, Hefei, Anhui 230026 China; 2 Suzhou Institute for Advanced Research, University of Science and Technology of China, Suzhou, Jiangsu 215123 China; 3 School of Engineering Science, University of Science and Technology of China, Hefei, Anhui 230026 China; 4 Department of Urology, the First Affiliated Hospital of Anhui Medical University, Hefei, Anhui 230031 China

**Keywords:** Electrical and electronic engineering, Engineering

## Abstract

Erectile dysfunction (ED) is a prevalent type of sexual dysfunction, and continuous monitoring of penile tumescence and rigidity during spontaneous nocturnal erections is crucial for its diagnosis and classification. However, the current clinical standard device, limited by its active mechanical load, is bulky and nonwearable and strongly interferes with erections, which compromises both monitoring reliability and patient compliance. Here, we report a wearable adaptive rigidity monitoring (WARM) system that employs a measurement principle without active loads, allowing for the assessment of penile tumescence and rigidity through a specifically designed elastic dual-ring sensor. The dual-ring sensor, comprising two strain-sensing rings with distinct elastic moduli, provides high resolution (0.1%), robust mechanical and electrical stability (sustaining over 1000 cycles), and strong interference resistance. An integrated flexible printed circuit (FPC) collects and processes sensing signals, which are then transmitted to the host computer via Bluetooth for ED assessment. Additionally, we validated the WARM system against the clinical standard device using both a penile model and healthy volunteers, achieving high consistency. Furthermore, the system facilitates the continuous evaluation of penile erections during nocturnal tumescence tests with concurrent sleep monitoring, demonstrating its ability to minimize interference with nocturnal erections. In conclusion, the WARM system offers a fully integrated, wearable solution for continuous, precise, and patient-friendly measurement of penile tumescence and rigidity, potentially providing more reliable and accessible outcomes than existing technologies.

**Figure:**
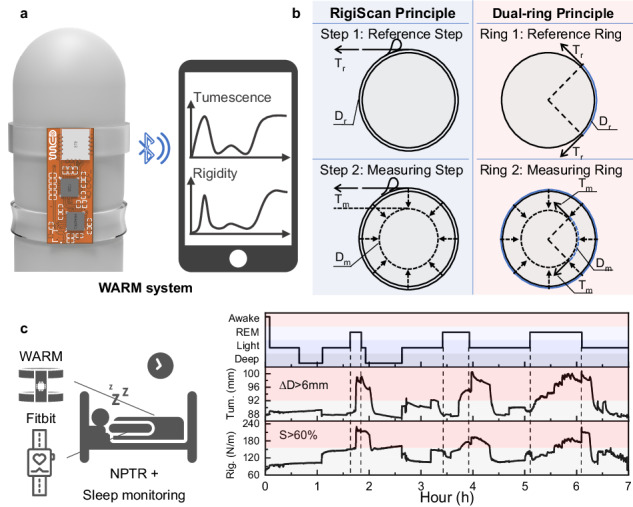
Erectile dysfunction (ED) is a prevalent sexual dysfunction, and continuous monitoring of penile tumescence and rigidity during spontaneous nocturnal erections is crucial for its diagnosis and classification. However, the current clinical standard device, limited by its active mechanical load, is bulky, nonwearable, and creates pronounced interference with erections, which compromises both monitoring reliability and patient compliance. Here, we report a wearable adaptive rigidity monitoring (WARM) system (Fig. 1a) that employs a measurement principle without active loads (Fig. 1b), allowing for the assessment of penile tumescence and rigidity through a specifically designed elastic dual-ring sensor. The dual-ring sensor, comprising two strain-sensing rings with distinct elastic moduli, provides high resolution (0.1%), robust mechanical and electrical stability (sustaining over 1000 cycles), and strong interference resistance. Additionally, we validate the WARM system against the clinical standard device using both a penile model and healthy volunteers, achieving high consistency. Furthermore, the system facilitates the continuous evaluation of penile erections during nocturnal tumescence tests, with concurrent sleep monitoring, demonstrating its ability to minimize interference with nocturnal erections (Fig. 1c). In conclusion, the WARM system offers a fully integrated, wearable solution for continuous, precise, and patient-friendly measurement of penile tumescence and rigidity, potentially providing more reliable and accessible outcomes than those from existing technologies.

Erectile dysfunction (ED) is a prevalent sexual dysfunction, and continuous monitoring of penile tumescence and rigidity during spontaneous nocturnal erections is crucial for its diagnosis and classification. However, the current clinical standard device, limited by its active mechanical load, is bulky, nonwearable, and creates pronounced interference with erections, which compromises both monitoring reliability and patient compliance. Here, we report a wearable adaptive rigidity monitoring (WARM) system (Fig. 1a) that employs a measurement principle without active loads (Fig. 1b), allowing for the assessment of penile tumescence and rigidity through a specifically designed elastic dual-ring sensor. The dual-ring sensor, comprising two strain-sensing rings with distinct elastic moduli, provides high resolution (0.1%), robust mechanical and electrical stability (sustaining over 1000 cycles), and strong interference resistance. Additionally, we validate the WARM system against the clinical standard device using both a penile model and healthy volunteers, achieving high consistency. Furthermore, the system facilitates the continuous evaluation of penile erections during nocturnal tumescence tests, with concurrent sleep monitoring, demonstrating its ability to minimize interference with nocturnal erections (Fig. 1c). In conclusion, the WARM system offers a fully integrated, wearable solution for continuous, precise, and patient-friendly measurement of penile tumescence and rigidity, potentially providing more reliable and accessible outcomes than those from existing technologies.

## Introduction

Erectile dysfunction (ED) is one of the most common male sexual dysfunctions and is characterized by the inability to achieve or maintain sufficient penile tumescence and rigidity for satisfactory sexual activities^1,2^. ED can severely affect the quality of emotional well-being of patients and their families. Notably, it is also an emerging marker for future cardiovascular diseases (CVDs)^3–5^. In recent years, the global incidence of ED has significantly increased due to factors such as environmental pollution, alterations in dietary patterns, and the impact of the COVID-19 pandemic^6,7^. Global incidence is predicted to reach 320 million cases by 2025^8^. Clinically, ED is mainly divided into organic and psychological types^1–3^. The former is commonly caused by physiological factors, including abnormalities or injuries to the corpus cavernosum, nervous system, secretory systems, and especially the cardiovascular system^9,10^. The latter is related to depression, mental stress, and environmental factors^11^. These two distinct types of ED require entirely different treatment methods and reveal different health risks^1–3^. Therefore, making an accurate diagnosis of ED and distinguishing its etiology are crucial for disease management and early warning of CVD.

Currently, the gold standard for ED diagnosis is the nocturnal penile tumescence and rigidity (NPTR) test^12,13^, which monitors penile rigidity and tumescence during spontaneous erection events in rapid eye movement (REM) sleep^14,15^. The NPTR test is designed to eliminate interference from psychological factors on erectile function, thus facilitating accurate diagnosis of ED and differentiation of its causes^12,16^. Clinically, the RigiScan device, introduced in the 1980s, has become the widely recognized noninvasive monitoring standard for NPTR testing^17,18^. Specifically, RigiScan measures tumescence and rigidity by alternately applying two different tangential stretches to the penis using a torque motor to drive penile loop contraction. Typically, during the NPTR test, patients are instructed to wear the RigiScan penile loop throughout their sleep at the hospital. As they sleep, the device’s torque motor alternately stretches the penile loop at short intervals (i.e., four times per minute), allowing for continuous monitoring and assessment of the patient’s nocturnal spontaneous erection events.

However, RigiScan, due to its dynamic mechanical measurement principle, relies heavily on intricate mechanical control to periodically drive the torque motor, leading to several challenges in its clinical applications^19–21^. Notably, the primary limitation of the RigiScan device is the size and weight of its torque motor and mechanical control system^22^. These factors limit the potential for reducing the overall dimensions and weight of the device, making it unsuitable in terms of wearability and limiting its use predominantly to hospital settings^21,23^. Additionally, the periodic operation of the torque motors generates significant operational noise and exerts a considerable stretching force of up to 2.78 N (10 ozf) on the penis. Such limitations can lead to patient discomfort and interfere with nocturnal spontaneous erections, which increases the risk of misdiagnosis and reduces patient compliance during the NPTR test, potentially compromising clinical accuracy and effectiveness^19,24,25^.

In recent years, significant advances in flexible electronics have propelled the rapid growth of wearable medical devices, making it possible to offer patients more convenient and comfortable health care services^26^. However, in the field of ED diagnosis, research on this topic remains scarce. For instance, Yoon and his team^27^ as well as Jo and his team^28^ have independently utilized flexible sensors to measure penile tumescence; however, these studies did not assess penile rigidity. In another study, Bhagat and his team evaluated penile rigidity using flexible strain sensors, but unfortunately, their work was limited to qualitative studies due to a lack of theoretical analysis of the measurements^29^. Therefore, there is still a demand for a precise, reliable, and patient-friendly method to simultaneously measure penile tumescence and rigidity.

Here, we report the first wearable adaptive rigidity monitoring (WARM) system designed to continuously measure both penile rigidity and tumescence for clinical ED assessment while concurrently minimizing interference with erections. To realize such functions, we have introduced a novel differential elastic measuring principle, termed the dual-ring method, to replace the conventional two-step force‒displacement process, thereby addressing the challenges of measurement reliability and patient compliance associated with periodic mechanical loading. Specifically, the dual-ring method is implemented using a highly elastic dual-ring sensor comprising two strain-sensing rings with different elastic moduli. One ring, with a low elastic modulus, calibrates the penile circumference, while the other, with a high elastic modulus, evaluates penile ability to resist deformation under radial compression. This sensor, which features a 7-layer parallel plate structure with each layer composed of an elastic polymer material, offers high spatial resolution (0.1%), robust mechanical and electrical stability (over 1000 stretch cycles), and strong interference resistance. An integrated flexible printed circuit (FPC) collects and processes sensor signals in real time and transmits them to the host computer via Bluetooth for display and ED assessment. Additionally, the WARM system was validated and characterized on both a penile model and healthy volunteers, demonstrating high measurement accuracy comparable to that of the gold standard (R^2^ > 0.98). Furthermore, we demonstrated the clinical utility of the WARM system by continuously monitoring the penile erections of healthy volunteers during audio-visual sexual stimulation (AVSS) and nocturnal penile tumescence and rigidity (NPTR) tests, with the latter accompanied by an assessment of sleep stages. The experimental results highlight the ability of the system to minimize interference with nocturnal erections. In conclusion, the WARM system offers a fully integrated, wearable solution for the continuous, accurate, and patient-friendly measurement of penile rigidity and tumescence for clinical-grade ED assessment, potentially leading to more reliable and accessible diagnostic outcomes than those from currently available technologies.

## Results

### Penile tumescence and rigidity measurement principle

**Fig. 1:**
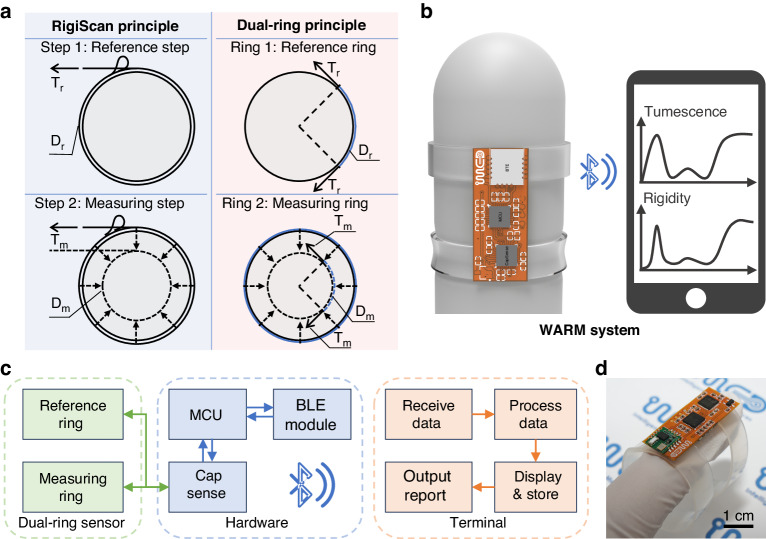
Wearable adaptive rigidity monitoring (WARM) system. **a** Comparison of the working principles between the RigiScan and WARM systems. The RigiScan employs a two-step process for measuring penile tumescence and rigidity. In contrast, the WARM system utilizes two strain-sensing rings with different elastic moduli for these measurements. **b** Schematic of the WARM system. **c** Block diagram of the WARM system, which includes a dual-ring sensor for penile tumescence and rigidity assessment, a flexible printed circuit for signal acquisition, processing and wireless transmission, and a terminal for data display and analysis. **d** Optical image of the WARM system

First, the working principle of the WARM system is compared to that of RigiScan, a widely recognized clinical device. The principle employed by the RigiScan for assessing penile tumescence and rigidity is based on dynamic mechanical measurements^30^. Specifically, assessed tumescence reflects changes in penile circumference during erection, and assessed rigidity reflects the ability to resist radial deformation under a known compression force^31^, as shown in Fig. 1a. It can be observed that RigiScan applies a tensile force in the tangential direction. Such tangential stretch can then be converted into radial compression, from which the radial shape of the penis should be altered with the corresponding circumference measured. To implement such a measurement, RigiScan employs a two-step process. Initially, a gentle reference load (*T*_r_) is periodically applied to measure the circumference (*D*_r_) of the penis without causing appreciable deformation. When a substantial change in penile circumference is detected, the RigiScan device can then apply a more robust constricting load (*T*_m_) while continuously evaluating the corresponding circumferential displacements (*D*_r_-*D*_m_), from which the radial rigidity (*S*) can be determined as *S* = (*T*_m_-*T*_r_)/(*D*_r_-*D*_m_)^17^. Consequently, a motorized loading component with a high-precision length-measuring unit must be included in the system design, which increases the system’s complexity and limits its usage in clinical settings^22,32^.

In this work, the principles of tumescence and rigidity adhere to a concept similar to that of RigiScan, with a focus on penile circumference and radial rigidity. However, a passive measurement technique, named the dual-ring method, has been applied, in which an elastic dual-ring sensor with elongation-measuring capacity is used instead of dynamic stretching loads. Notably, the measuring ring sensor offers two relevant parameters in the measurements: the linear tension (*T*_m_), which can be converted into radial compression, and the corresponding circumferential distance (*D*_m_). These typically adhere to Hooke’s law *T*_m_ = (*E*·*A*/*D*_0_) · (*D*_m_-*D*_0_), where *E*, *A*, and *D*_0_ represent the elastic modulus, the cross-sectional area, and the initial circumference of the elastic ring, respectively^33^. To decouple such relevant force‒displacement measurements, a reference ring adjacent to the measuring ring is introduced. It possesses a lower modulus of elasticity than does the penis, so it does not cause appreciable deformation when applied and can provide a reference value (*D*_r_) for penile tumescence^22^. Thus, rigidity can be calculated using the same definition as mentioned above. In brief, the reference ring offers circumferential measurement, while the measuring ring contributes to the force–displacement assessment, equivalent to the two-step dynamic measurement of RigiScan. Through such differential measurements, the WARM system can perform continuous assessments of both tumescence and rigidity without any moving parts during the erection process.

### Design of the WARM system

Figure 1b presents a schematic diagram of the WARM system, which is designed for monitoring penile erection. The wearable sensing device provides real-time information on penile rigidity and tumescence, which is transmitted instantly to mobile devices via a Bluetooth module, facilitating more precise analysis and diagnosis by doctors. As shown in Fig. 1c, the system comprises three primary components: a precise and stable dual-ring sensor consisting of two sensing units with different elastic moduli, namely, reference and measuring rings; a flexible printed circuit (FPC) integrated with a capacitance detection chip, microcontroller unit (MCU), and Bluetooth module (Figs. S1–3); and a terminal responsible for data display, storage, and analysis (Fig. S4). Specifically, the flexible dual-ring sensor, with its distinct elastic moduli, can adaptively apply varying load levels to the penis and translate circumferential data into capacitance outputs. The sensor connects reversibly to the FPC board via a standard connector, facilitating signal acquisition by the capacitance detection module. Subsequently, the MCU conducts real-time evaluations of penile rigidity and tumescence. Once assessed, the results are transmitted to the mobile terminal via Bluetooth, where the data are processed and analyzed. The WARM system features low power consumption and is capable of operating continuously at a measurement frequency of 1 Hz for up to 10 h, ensuring compliance with NPTR test time requirements (Fig. S5).

Figure 1d presents the WARM system prototype. By utilizing passive measurements of rigidity and tumescence, the system achieved more compact size, reduced weight, and simplified design compared to RigiScan (Fig. S6). The dual-ring approach allows the elastic sensor to apply radial compression and provide continuous circumferential measurements simultaneously. In contrast, RigiScan uses a torque motor to apply dynamic tensile loads to the penis and a potentiometer to record changes in penile circumference, which increases the system complexity (Fig. S7)^17^.

### Design of the elastic dual-ring sensor

As mentioned previously, the measurement principle of the WARM system requires continuous evaluation of penile circumference and circumferential displacement under known loads. Thus, an elastic dual-ring sensor based on a flexible capacitive strain sensor design has been implemented for this purpose. First, the elastic strain sensor is used to assess circumferential changes during the tumescence process, converting surface elongation into electrical signal readouts^29,34^. Second, the strain sensor contains two sensing rings with different elastic moduli, constituting an elastic dual-ring structure, allowing the sensor design to be easily customized for the desired length and mechanical performance using the same processing flow. As a result, we can implement dual-ring measurements: one for penile circumference with low elastic moduli and the other for circumferential displacement with high elastic moduli. Moreover, the strain sensor must be highly mechanically repeatable and reliable, a critical feature for continuous measurements that involve multiple tumescence and detumescence cycles. In addition, its electrical stability and anti-interference performance are essential for evaluating measurements through multiple tumescence and detumescence cycles for different patients, each potentially lasting 10 minutes or longer, thereby demanding accurate readouts without drifting^12,18^.

**Fig. 2:**
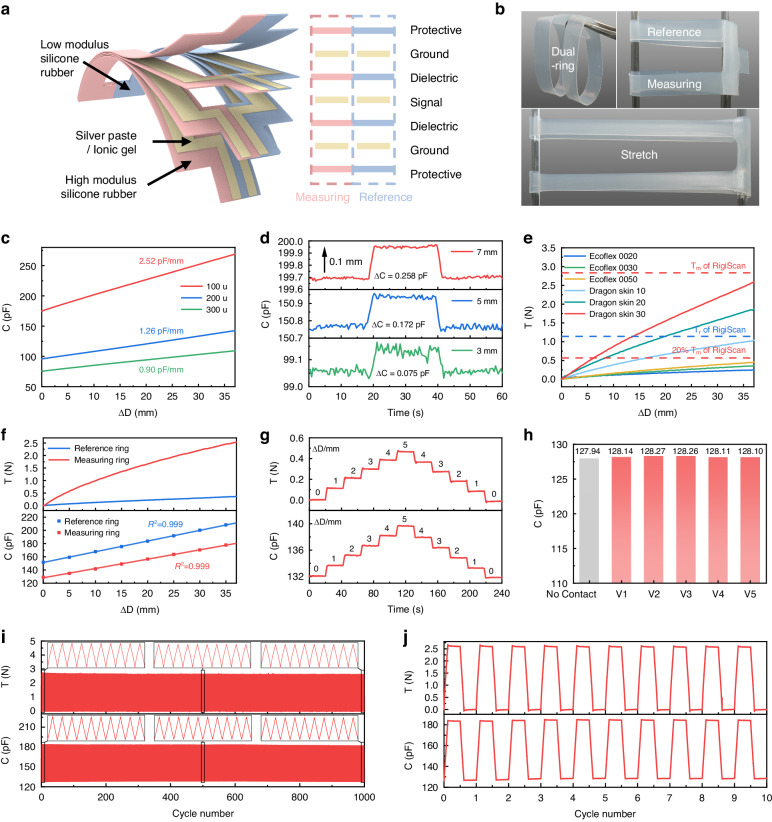
Design and characterization of the dual-ring sensor. **a** Planar layout of the dual-ring sensor, designed with a 7-layer parallel-plate sensing architecture. **b** Optical images of the dual-ring sensor. **c** Effect of the dielectric layer thickness on the electrical performance of the sensor, given a fixed electrode layer width. **d** Capacitance responses of the sensor with electrode layer widths of 3 mm, 5 mm, and 7 mm when subjected to 0.1 mm deformation. **e** Mechanical performances of samples made from various silicone rubbers. The design specifications require the measuring ring’s maximum tension to be less than the force used by RigiScan for rigidity measurement (*T*_m_), with the reference ring’s maximum tension being less than 20% of this value. **f** Capacitance-circumferential displacement and tension-circumferential displacement responses for the reference and measuring rings. **g** Capacitance and tension recordings for the measuring ring when subjected to stepped 1 mm deformation. **h** Comparison of the initial capacitance of the measuring ring in both the noncontact and contact states across five volunteers. **i** Mechanical and electrical performance of the measuring ring during 1000 tensile cycles. Inset: Performance during the 1st-10th, 496th-505th, and 991st-1000th cycles. **j** Mechanical and electrical performance of the measuring ring during cyclic stretching, holding for 10 minutes, relaxing, and then holding for another 10 minutes

Figure 2a presents the planar layout of the elastic dual-ring sensor, which consists of reference and measuring rings, both of which are based on the parallel-plate capacitive principle for strain measurement. The parallel-plate sensing architecture has been proven to be highly mechanically and electrically stable for continuous measurements^35,36^. It is designed as a 7-layer structural assembly in which all layers are made from highly elastic polymeric materials, and the electrically conductive layers are always separated by adjacent insulating layers. The middle conductive layer serves as the main signal transmission layer, while the two external conductive layers are electrically connected. The capacitances between the middle conductive layer and the two external layers are both measured for the assessment of penile tumescence and rigidity, and importantly, the two external layers form a Faraday shield by connecting to the ground to prevent environmental electromagnetic influences^37^. This structural design also leads to increased initial capacitance and device sensitivity by doubling the capacitive overlapping area. The conductive layers can be produced using highly conductive and elastic ionic gels or commercial stretchable conductive silver paste, while the protective and dielectric layers can be made from readily available silicone rubber. Due to the fully flexible design, the overall structure of the sensor can withstand bending and stretching without mechanical fracture (Fig. 2b). To implement the dual-ring principle for measuring rigidity and tumescence, key design parameters, such as initial circumference, maximal circumferential displacement, and the corresponding maximal tensile load, must be defined for the dual-ring sensor. The detailed design principles can be found in Note S1.

### Optimization and characterization of the elastic dual-ring sensor

The optimal geometric parameters of the dual-ring sensor, including the thickness of the dielectric layer and the width of the electrode layer, need to be determined to meet the specific mechanical and electrical requirements of the design principle. Additionally, it is essential to select suitable materials for each layer.

Initially, the influence of the dielectric layer thickness on the electrical performance of the dual-ring sensor was studied with a fixed electrode layer width of 3 mm. A thinner dielectric layer enhances both the sensitivity and initial capacitance of the sensor^38^; however, it also increases the manufacturing difficulty and the risk of electrical failure (Fig. S8). After evaluation, a dielectric layer thickness of 200 μm was chosen for this work. This thickness offers an optimal balance between sensitivity and reliability, as illustrated in Fig. 2c, with a sensitivity and initial capacitance of 95.86 pF and 1.26 pF/mm, respectively. Furthermore, to achieve the desired precision in penile circumference measurement, the sensor must exhibit significant and stable capacitance changes with a deformation of 0.1 mm, which approximately corresponds to 0.1% of the sensor strain. As illustrated in Fig. 2d, samples with electrode widths of 5 mm and 7 mm show stable capacitance increases of 0.172 pF and 0.258 pF, respectively, with a 0.1 mm deformation. Moreover, a narrower sensor is preferred to minimize nonuniform deformation in the width direction of the sensor. Hence, the recommended parameters for the dual-ring sensor are a dielectric layer thickness of 200 μm and an electrode layer width of 5 mm.

After determining the geometric dimensions of the sensor, it is essential to select appropriate materials to ensure that the mechanical performance meets the design requirements. Silicone rubber samples with varying elastic moduli were prepared and tested. The Ecoflex and Dragon Skin silicone rubber series were selected because of their significantly lower moduli than those of other insulating elastomers (Table S1). The test results, as illustrated in Fig. 2e, demonstrate considerable variation in the maximum tensile load of the samples with different silicone rubbers, ranging from 0.248 N to 2.590 N. Following the design principle of the dual-ring sensor, Ecoflex 0020 and Dragon Skin 30 were chosen as the insulating layer materials for the reference and measuring rings, respectively. Specifically, Ecoflex 0020 and Dragon Skin 30 exhibit the minimum and maximum elastic moduli, with maximum tensile loads of 0.248 N and 2.590 N, respectively, both of which are below the design’s upper limits. Furthermore, the influence of sample thickness on the maximum tensile load is detailed in Fig. S9. Additionally, both ionic gel and conductive silver paste have been tested as electrode layer materials and have maintained good conductivity over 1000 tensile cycles (Fig. S10); thus, both materials are suitable as electrode layers. Ionic gels are easy to prepare, cost-effective, and transparent, while conductive silver paste offers lower resistance. With these material choices for insulating and electrode layers, the dual-ring sensor meets the design requirements in terms of mechanical performance, wearing comfort, and electrical conductivity.

After optimization, the dual-ring sensor was fabricated and characterized to assess its mechanical and electrical properties. Detailed information on the fabrication and characterization procedures is available in the Methods and Materials section and in Figs. S11–12. Figure 2b and Fig. S13 display optical images of the sensors fabricated using the ionic gel and conductive silver paste, respectively. Figure 2f shows the capacitance-circumferential displacement and tension-circumferential displacement responses of the reference and measuring rings. Even when stretched by the maximum designed circumferential displacement of 37 mm, the observed maximum tensile forces for the reference and measuring rings were 0.365 N and 2.528 N, respectively, which are lower than the 1.11 N (4 ozf) and 2.78 N (10 ozf) associated with RigiScan. Reducing measurement loads results in less interference with erections. Moreover, the reference and measuring rings offer a distinct advantage by adaptively increasing tensile loads during erection while significantly reducing them in the flaccid state, as opposed to the constant load employed by RigiScan. The adaptive load of the dual-ring sensor is more conducive to continuous and imperceptible monitoring of penile rigidity and tumescence. Additionally, the capacitance of both rings shows a highly linear relationship with the circumferential displacement. By continuously measuring the capacitance of the dual-ring sensor, the circumferences of the reference and measuring rings (*D*_r_ and *D*_m_) can be determined in real time. These values are then used to compute the tensile loads (*T*_r_ and *T*_m_) exerted by the rings, thereby completing the calculation of penile rigidity (*S*) and tumescence (*D*_r_).

Figure 2g presents the capacitance and tension recordings of the sensor using the measuring ring, which was subjected to stepped changes of 1 mm deformation. The results indicate that the capacitance and tensile loads of the sensor undergo significant changes with each small deformation and remain stable for the specified durations. In Fig. 2h, the initial capacitance of the measuring ring is compared between the noncontact and contact states across five volunteers. The initial capacitance values exhibit minor differences (below 0.33 pF), suggesting that parasitic capacitance variations from different patients will not significantly influence the practical measurements of the sensor.

The repeatability of the elastic dual-ring sensor was evaluated through tensile cycle tests on both rings. Figure 2i and Fig. S14 illustrate the consistent mechanical and electrical performance of the measuring and reference rings, respectively, with no noticeable baseline drift after 1000 cycles. To assess its reliability in practical applications, a motorized force tester was used to replicate the stages of penile tumescence, maintenance, and detumescence while monitoring the mechanical and electrical behavior of the sensor. Figure 2j and Fig. S15 indicate that both rings maintain stable mechanical and electrical properties during the 10-minute maintenance phase, with no pronounced stress relaxation or capacitance shifts. This stability can be attributed to the parallel plate capacitance design of the sensor. In contrast, the electrical signal of a resistive strain sensor can be significantly disrupted by irreversible changes in the conductive network during cycling, thereby compromising its electrical cycle stability (Fig. S16)^39^.

### Feasibility testing of the WARM system

**Fig. 3:**
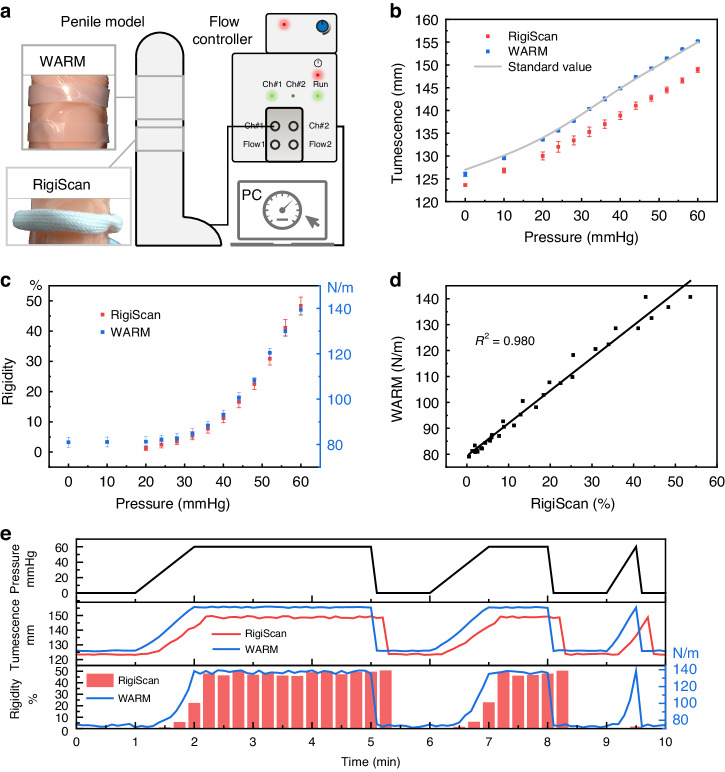
Feasibility testing of the WARM system. **a** Penile tumescence simulator consisting of an expandable penile model, a flow controller for adjusting the model rigidity and circumference, and a computer with the corresponding control software. **b** Comparison of the tumescence measurements of the expandable penile model at varying internal pressures using both the WARM system and the RigiScan (*N* = 3), benchmarked against standard measurements from a flexible measuring tape. **c** Comparison of the rigidity measurements of the expandable penile model at different internal pressures utilizing both the WARM system and the RigiScan (*N* = 3). **d** Linear regression analysis of rigidity measurements from both systems. The coefficient of determination R^2^ for the linear regression is 0.98. **e** Comparison of tumescence and rigidity measurements between the two systems during the simulated penile erection process. The internal pressure of the penile model is designed to increase from 0 to 60 mmHg at a rate of 1 mmHg/s and then remain for 3 min, 1 min, or 0 min before decreasing rapidly to 0 mmHg

A penile tumescence simulator has been developed to efficiently characterize the WARM system. It allows for stable and programmable adjustments of the rigidity and tumescence of an expandable penile model, providing a standardized testing platform to evaluate the WARM system. Figure 3a presents the simulator, which comprises an expandable penile model, a flow controller for indirectly adjusting the model’s rigidity and circumference, and a computer equipped with corresponding control software. The penile model, a commercially available hollow circular cylinder made of elastic silicone, reflects the physiological characteristics of penile rigidity and tumescence that vary with intracavernosal pressure^40,41^. By modulating its internal pressure, the rigidity and tumescence of the model can be flexibly controlled. With the pressure control unit (i.e., the flow controller) connected, precise adjustments to the rigidity and tumescence of the model can be programmed through the computer.

The tumescence measurements of the expandable penile model at varying internal pressures, as evaluated by both the WARM system and the RigiScan, are presented in Fig. 3b, along with standard measurements obtained using a flexible measuring tape. As anticipated, the tumescence of the model correlates with its internal pressure. The measurements obtained by the WARM system align closely with the standard values, exhibiting a maximum deviation of less than 1 mm. Conversely, the RigiScan consistently yields lower values, with discrepancies of up to 6.4 mm, likely due to its excessive tensile load of 1.11 N causing significant compression deformation of the model^42^. The WARM system, employing a reference ring with ultralow elastic moduli, achieves more accurate measurements while applying only a maximal tensile load of 0.24 N. Additionally, the WARM system’s mean standard deviation for tumescence measurements is 0.269, which is lower than that of RigiScan (0.751), indicating better repeatability.

Figure 3c presents a comparison of rigidity measurements obtained from both the WARM system and the RigiScan on the penile model under varying internal pressures. Notably, the RigiScan quantifies rigidity as a percentage relative to a specific hard rubber cylinder^17^. Additionally, to enhance measurement comfort, RigiScan applies a robust tensile load of 2.78 N only when significant circumferential changes are detected^17,22^. Consequently, RigiScan measurements of rigidity are initiated only when the penile model reaches an internal pressure of 20 mmHg. This restriction compromises the ability of RigiScan to continuously monitor erection. In contrast, the WARM system, due to the adaptive tensile load applied by the elastic dual-ring sensor, maintains the tensile load for rigidity measurements below 1 N when the internal pressure of the model is below 20 mmHg. This adaptability enables the WARM system to monitor penile rigidity continuously in a patient-friendly manner without requiring full erection.

The linear regression analysis of rigidity measurements from both systems in Fig. 3d demonstrates a high level of consistency, yielding a correlation coefficient of 0.98. This strong linear relationship is anticipated due to the shared definition and calculation formula for the radial rigidity employed by both the WARM system and the RigiScan. However, their measuring principles differ: while the WARM system utilizes elastic dual-ring measurements, RigiScan adopts the conventional two-step force‒displacement process^17^.

To mimic the process of penile tumescence-detumescence during actual erection monitoring, the penile model was subjected to multiple cycles of inflation and deflation of varying durations. Both the RigiScan and WARM systems monitor this process simultaneously, and the results are presented in Fig. 3e. A comparison of these results reveals that the WARM system offers real-time and continuous assessment of penile tumescence and rigidity, exhibiting advantages such as rapid response, more accurate circumference measurement, and wireless data transmission. Specifically, the curves for tumescence and rigidity, as measured by the WARM system, align synchronously with the internal pressure curve of the model; however, there are noticeable delays in the results measured by RigiScan. Notably, even during rapid inflation and deflation at 9.5 minutes, the WARM system accurately captured changes in penile tumescence and rigidity. In conclusion, the WARM system enables continuous and accurate measurement of penile tumescence and rigidity in a wearable, wireless, and patient-friendly manner.

### Assessment of penile erection in volunteers

**Fig. 4:**
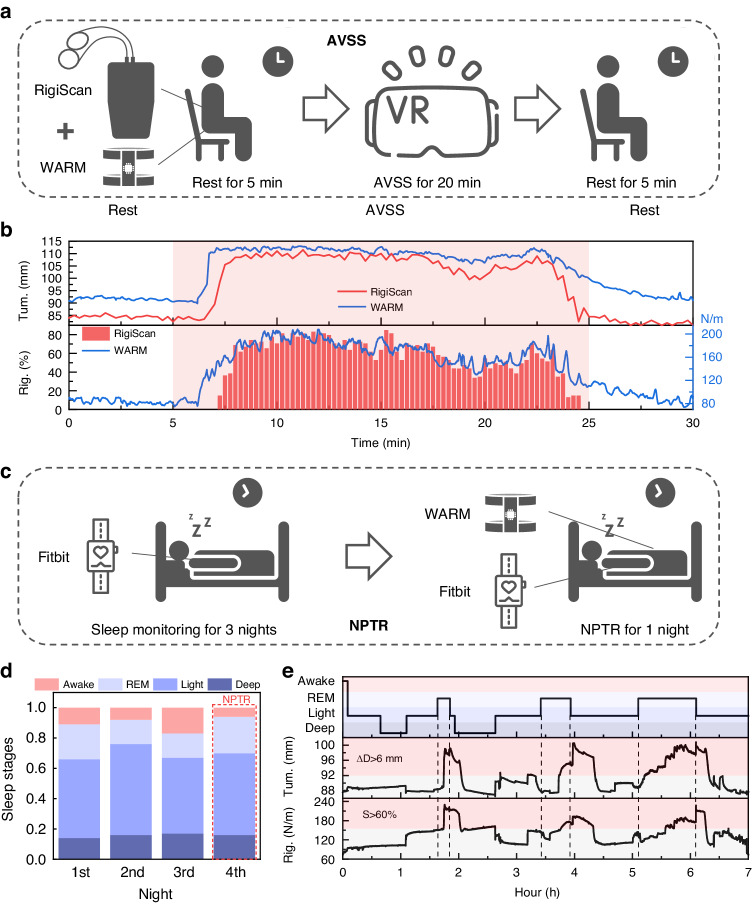
Volunteer testing with the WARM system for continuous penile erection assessments. **a** Procedure for the AVSS test using both the RigiScan and the WARM system as penile erection monitoring tools. **b** Comparison of tumescence and rigidity measurements between the two systems during the AVSS test. **c** Procedure for the NPTR test: Sleep was monitored for three consecutive nights, followed by a synchronized NPTR-sleep test on one night. **d** Sleep stage data for four consecutive nights as recorded by Fitbit. Sleep stages are categorized into deep sleep, light sleep, rapid eye movement (REM), and awake periods. During the fourth night, both the Fitbit and WARM systems were worn simultaneously for the NPTR test. The proportions of deep sleep and REM sleep observed were not significantly different from those of the previous three nights. **e** Sleep stages and penile tumescence and rigidity data during the fourth night. Regions where the penile circumference increased by more than 6 mm or where rigidity exceeded 60% are highlighted in red. On the one hand, the close relationship between nocturnal erection and REM was verified; on the other hand, the reliability of the WARM system was confirmed

To further evaluate the clinical utility of the WARM system in monitoring erections and diagnosing ED, two volunteers were recruited for the AVSS and NPTR tests. Currently, the AVSS and NPTR tests serve as the two primary diagnostic methods used to differentiate between psychological and organic ED because of their noninvasive and objective nature^43^. Specifically, the NPTR test is considered the gold standard due to its greater accuracy, while the AVSS test, which is less time-consuming, has gained increasing popularity for rapid clinical screening of impotence^12,44^. The results of the AVSS test are shown in Fig. 4a, and detailed information is available in Table S2. It can be observed that following 2 minutes of audio-visual stimulation, there was a significant increase in both tumescence and rigidity of the penis, signifying an erection. This erection persisted until minute 22.5, followed by a gradual decline in tumescence and rigidity, indicating the end of the erection. Comparative analysis reveales that the WARM system offers exceptional real-time tracking of penile erection status, delivering more precise circumferential assessments and high-precision rigidity measurements than the RigiScan system. Notably, the tumescence values recorded by the WARM system are marginally greater than those of the RigiScan system, and the rigidity curves from both systems exhibit nearly identical temporal variation characteristics. In clinical settings, using the RigiScan as an ED diagnostic tool, a patient is considered to have normal erectile function if their penile rigidity exceeds 60% (equivalent to 155 N/m as measured by the WARM system) for more than 8.75 minutes during the AVSS test^44^. The results presented in Table S2 demonstrate a consistent duration of penile rigidity exceeding 60%, as measured by both the RigiScan and the WARM systems, with values of 11 and 12 minutes, respectively, confirming the subject’s normal erectile function.

The clinical value of the WARM system has been further confirmed through the NPTR test. Nocturnal penile tumescence, a normal physiological phenomenon, typically occurs during rapid eye movement (REM) sleep^45^. Undisturbed, healthy males typically experience 3-5 spontaneous erections per night, with at least one achieving rigidity exceeding 60% and persisting for more than 10 minutes^12,46^. However, the discomfort induced by current erection monitoring devices may interrupt the subject’s sleep and the associated spontaneous erections^24^, potentially yielding false-positive diagnostic results^19,25^. To confirm the WARM system’s ability to minimize interference with sleep and nocturnal erections, the second volunteer initially wore a Fitbit for continuous sleep monitoring over three nights. On the fourth night, he was instructed to wear both the Fitbit and the WARM systems for a combined NPTR-sleep assessment (Fig. 4c). The Fitbit, a commonly used wearable sleep monitor in medical research, offers insights into sleep stages^15,47^. As illustrated in Fig. 4d, when exclusively wearing the Fitbit, the proportions of the subject in the deep sleep, light sleep, REM, and awake stages for the first three nights were found to be 0.16 ± 0.02, 0.54 ± 0.05, 0.18 ± 0.04, and 0.12 ± 0.05, respectively. On the fourth night with simultaneous use of both devices, these proportions were 0.16, 0.54, 0.24, and 0.06, respectively. The data indicate that the implementation of the WARM system does not compromise sleep quality since there is no observed reduction in either deep sleep or REM phase duration, while there is also no increase in the awake phase. Figure 4e provides a detailed analysis of sleep data and NPTR outcomes, emphasizing regions where penile circumference increased by more than 6 mm or where rigidity exceeded 60% in red. The sleep stage graph reveals three REM episodes at 1.63 hours, 3.41 hours, and 5.1 hours after the onset of sleep, lasting for 13, 31, and 60 minutes, respectively. The tumescence chart indicates three pronounced erections (tumescence over 6 mm) at 1.74 hours, 3.73 hours, and 5.22 hours, lasting for 18, 37, and 66 minutes, respectively. These erections, which initiated approximately 5–20 minutes after the onset of REM sleep, lasted for durations similar to those of REM phases. This comparison substantiates the close association between nocturnal erections and REM sleep and validates the reliability of the WARM system for monitoring the NPTR. The rigidity curve also indicates three notable erections with rigidities above 60%, lasting for 18, 34, and 42 minutes, respectively. Each erection persisted for more than 10 minutes, verifying the volunteer’s healthy erectile function. In conclusion, during the seven-hour NPTR monitoring period, the WARM system demonstrates high fidelity and precision with minimal interference to erections, thereby enhancing its clinical value in diagnosing ED.

## Conclusions

In summary, we have introduced the wearable adaptive rigidity monitoring (WARM) system, which is designed to continuously monitor penile rigidity and tumescence for clinical ED diagnosis. Specifically, we have presented a differential elastic measuring principle by use of the dual-ring method to replace the conventional two-step force‒displacement process. This method allows for assessing penile erections through a specifically designed elastic dual-ring sensor, addressing the challenges of measurement reliability and patient compliance associated with mechanical moving parts. The dual-ring sensor, comprising two strain-sensing rings with distinct elastic moduli, provides high resolution (0.1%), robust mechanical and electrical stability (sustaining over 1000 cycles), and strong interference resistance. An integrated flexible printed circuit (FPC) collects and processes sensor signals in real time, transmitting them via Bluetooth to the terminal for ED assessment. To validate the reliability of the WARM system, we conducted exhaustive comparisons between the WARM system and RigiScan by performing experiments on both a penile model and human subjects. The findings revealed that the WARM system can proficiently monitor penile rigidity and tumescence, offering accuracy comparable to that of standard clinical devices (R^2^ > 0.98) while ensuring minimal interference with erections. In conclusion, the proposed WARM system delivers a fully wearable, integrated solution that continuously and accurately measures penile rigidity and tumescence in a patient-friendly manner, potentially leading to more reliable and accessible outcomes in the clinical diagnosis of ED.

## Materials and methods

### Materials and reagents

Chitosan (C_6_H_11_NO_4_) _n_, gelatin, glycerol (C_3_H_8_O_3_), glacial acetic acid (CH_3_COOH), and sodium chloride (NaCl) were purchased from Aladdin. Ecoflex 0020, Ecoflex 0030, Ecoflex 0050, Dragon Skin 10, Dragon Skin 20, Dragon Skin 30, Ease Release 200 and Sil-Poxy were purchased from Smooth-On. Stretchable conductive silver paste was purchased from Shanghai Julong Electronic Technology Co., Ltd.

### Ionic gel fabrication

The ionic gel used as the conductive layer of the sensor was prepared as follows: First, 0.8 g of NaCl, 1.2 g of chitosan, and 300 μL of glacial acetic acid were combined in 30 mL of deionized water. The mixture was then stirred magnetically for 2 hours until the chitosan fully dissolved. Next, 1.2 g of gelatin was added to the solution. The solution was heated and maintained at 50 °C while being stirred magnetically until the gelatin was completely dissolved. Subsequently, 6 g of glycerol was added, and the solution was stirred magnetically for an additional 30 minutes. The prepared ionic gel was then placed in a centrifuge at 5000 rpm for 5 minutes to eliminate bubbles. Finally, the ionic gel was sealed and stored at 50 °C for later use.

### Dual-ring sensor fabrication

The elastic dual-ring sensor is designed as a 7-layer structural assembly based on the parallel-plate capacitive principle. As shown in Fig. S11, the entire manufacturing process of the sensor is straightforward, achieved by repetitively using the standard blade-coating technique. Specifically, the fabrication process involves the following steps: Step 1: A 500 μm thick insulating layer with distinct elastic moduli on the left and right sides was prepared on an iron plate using Ecoflex 0020 and Dragon Skin 30. Then, the silicone rubber precursors were cured at 60 °C for 1 h in a vacuum drying oven. Step 2: A PET film was laser-cut into the desired electrode pattern, and then a 100 μm thick patterned conductive layer was prepared on top of the insulating layer with the assistance of the PET film. As a conductive layer material, silver paste or ionic gel needs to be cured at 150 °C or at room temperature for 30 minutes, respectively. When using an ionic gel, plasma treatment on the surface of the insulating layer is required to improve the coating effect. Step 3: Steps 1 and 2 were repeated. The insulating and conductive layers were alternated until a 7-layer sensing structure was formed. It should be noted that the thickness of the dielectric layer was 200 μm, while that of the protective layer was 500 μm. Step 4: The composite film was laser-cut into the pattern of the dual-ring sensor and then bound into a ring using a stretchable silicone adhesive (Sil-Poxy). Step 5: The dual-ring sensor was connected to a standard FPC connector (65801-004LF, Amphenol ICC, USA).

### Mechanical characterization

The mechanical properties of different types of silicone rubber (Ecoflex 0020, Ecoflex 0030, Ecoflex 0050, Dragon Skin 10, Dragon Skin 20, and Dragon Skin 30) were tested to determine the most appropriate type for fabricating the dual-ring sensor. The samples were cut into uniform sizes (100 × 7 × 1.5 mm) and subjected to tensile tests using an ESM303 motorized force tester (Mark-10, USA). The initial length, elongation, and deformation rate of the sample were set to 79 mm, 37 mm, and 60 mm/min, respectively. The variation in the force gauge reading with the sample length was recorded. The mechanical performance of the fabricated dual-ring sensor was tested using the same equipment and parameter settings. The mechanical repeatability of the dual-ring sensor was tested through 1000 tensile cycles. To further verify the stability of the sensor in practical applications, the ESM303 was used to simulate the process of penile erection and detumescence. The sensor was first stretched from 79 mm to 116 mm at a speed of 10 mm/min and then maintained for 10 minutes. Next, the sensor was recovered from 116 mm to 79 mm at the same speed and maintained for another 10 minutes. This process was repeated 10 times. The variation in the force gauge reading with the sample length was recorded.

### Electrical characterization

In the tensile tests and practical application simulation experiments, the capacitance changes of the sensor were measured using an E4980AL precision LCR meter (KEYSIGHT, USA). In addition, to establish a more accurate relationship between the capacitance of the dual-ring sensor and the circumference of the measured object, cylindrical models with different circumferences were 3D printed for sensor calibration (Fig. S12). The circumferences of the models were 79 mm, 84 mm, 89 mm, 94 mm, 99 mm, 104 mm, 109 mm, and 114 mm. The dual-ring sensor was looped over the models with different circumferences, and the corresponding capacitance of the sensor was recorded.

### Hardware fabrication

The hardware circuitry of the WARM system is primarily responsible for capacitance detection, data processing, and Bluetooth transmission. The core electronic components include a microcontroller unit (MCU, STM32 L051K8U6, STMicroelectronics), a capacitance-to-digital conversion chip (Pcap01, ACAM), and a Bluetooth module (BT24-T, Shenzhen Daxia Longque Technology). The Pcap01 chip is responsible for real-time measurement of the capacitance of the reference and measuring rings and sends the measurement results to the MCU through the SPI serial communication interface. The MCU calculates the circumference (*D*_r_ and *D*_m_) and tensile load (*T*_r_ and *T*_m_) of the reference and measuring rings and then evaluates the penile rigidity (*S*). The original and processed results are packaged and then sent to the Bluetooth module through a serial port.

### System evaluation

A testing platform is designed to evaluate the accuracy of the WARM system in measuring penile rigidity and tumescence. It consists of an expandable penile model, a flow controller (PreciGenome, USA) for adjusting the internal pressure of the model, and a computer with the corresponding control software installed (Fig. S17). Specifically, the penile model is stepwise inflated, and the rigidity and tumescence of the model at different internal pressures are measured using RigiScan (GOTOP, USA) and the WARM system, respectively. To evaluate the tumescence measurement error, a flexible measuring tape is used to remeasure the tumescence of the model. Furthermore, to simulate the process of penile tumescence-detumescence during actual erection monitoring, the penile model was inflated and deflated three times. Specifically, the internal pressure of the penile model increased from 0 to 60 mmHg at a rate of 1 mmHg/s, remained at 60 mmHg for 3 minutes, 1 minute, or 0 minutes, and then decreased rapidly to 0 mmHg. The rigidity and tumescence of the model were monitored simultaneously using both the RigiScan and the WARM systems during the aforementioned process.

### Volunteer validation

To demonstrate the potential clinical applications of the WARM system, volunteer tests were conducted. Two 27-year-old male participants were recruited for the AVSS and NPTR tests. All tests were performed under an IRB protocol approved by the University of Science and Technology of China (IRB no. YXLLSH-SQ-2023-08-01). In the AVSS test, the volunteer was placed in a clean and comfortable room without external interference, given a 5-minute rest followed by 20 minutes of audio-visual stimulation, and then given another 5 minutes of rest. Throughout the entire test period, the WARM system and RigiScan were used to simultaneously monitor the penile rigidity and tumescence of the volunteer. For the NPTR test, the volunteer was required to wear a Fitbit (Fitbit, USA) for three consecutive nights of sleep monitoring and then wear both the Fitbit and the WARM system for continuous 7-hour synchronized NPTR-sleep monitoring. After the completion of the AVSS and NPTR tests, statistical information about the changes in penile tumescence and rigidity, as well as the number and duration of erections, was collected to assist in the diagnosis of ED.

## Supplementary information

supplementary information

## Data Availability

The data supporting the findings of this work are available within this paper and the supporting information files. The data are available upon request from the corresponding authors.
