# Supplementary material for: A wearable adaptive penile rigidity monitoring system for assessment of erectile dysfunction
Source: Microsyst Nanoeng. 2024 Sep 20;10:131. doi: 10.1038/s41378-024-00721-5 (PMC11413319; doi:10.1038/s41378-024-00721-5)
Supplement: Supplementary file 1 — supplementary information [file 41378_2024_721_MOESM1_ESM.pdf]

Supplementary Information

A Wearable Adaptive Penile Rigidity Monitoring System for Erectile Dysfunction Assessment

Xiangyang Wang, Ruojiang Wang, Yuyang Zhang, You Wu, Xu Wu, Zihao Luo, Yu Chang, Xiansheng Zhang and Tingrui Pan*

Correspondence to:

tingrui@ustc.edu.cn (T. P.)

The file includes:

Note S1

Figs. S1-17

Tables S1-2

Note S1. Design of the Dual-Ring Sensor

To implement the dual-ring principle for rigidity and tumescence measurement, the key design parameters, including the initial circumference, the maximal circumference displacement, and the corresponding maximal tensile load, should be established for the dual-ring sensor. According to a recent epidemiological survey, the average natural flaccid circumference of the penis is 85.4 ± 6.2 mm for Chinese men, which increases by an average of 22.1 ± 14.7 mm when fully erected [1]. Therefore, to ensure the secure attachment of the dual-ring sensor during the entire measurement, the initial circumference of the dual-ring sensor is set at 79 mm, less than the average natural flaccid circumference minus one standard deviation, the size of which could fit 84% of the male population if the circumferential statistics follow a Gaussian distribution [2]. Moreover, the maximal circumferential displacement has been chosen to be 37 mm, greater than one standard deviation beyond the average circumferential increase for the same reason. The maximum circumferential displacement corresponds to the maximum tensile load. As aforementioned, the reference ring is designed for real-time measurement of the penile circumference, which is intended to cause minimal deformation of the penis. Thus, the reference ring is desired to possess high elasticity with a low value of the maximum tensile load. Unlike the reference ring, the measuring ring determines the force and displacement differences relative to the reference ring, and therefore, the maximal tensile load, achieved by high elastic moduli, should be considerably measurable in order to reduce potential measurement errors while providing reasonable comfort to the patients for the extended period. Therefore, we have set the standard load (2.78 N / 10 ozf) of the RigiScan as the maximal tensile load of the measuring ring of the WARM system, of which the maximal tensile load for the reference ring is designed to be 20% (0.556 N / 2 ozf).

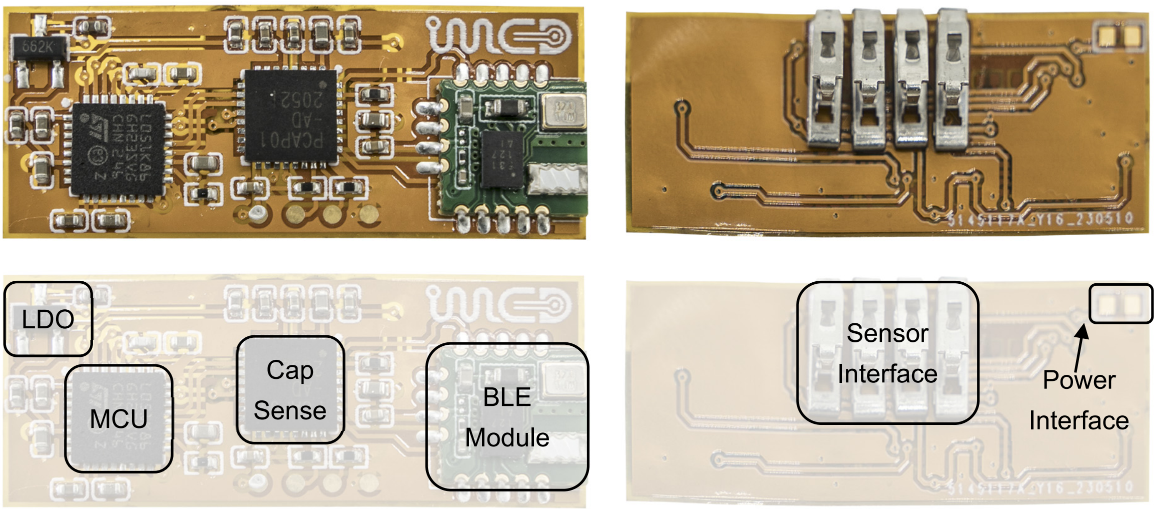

Fig. S1 Optical images of the Flexible Printed Circuit (FPC) for the WARM system. The front of the FPC features a Low Dropout Regulator (LDO), an MCU, a capacitor detection chip, and a Bluetooth module. The back of the FPC houses a sensor interface and a power interface.

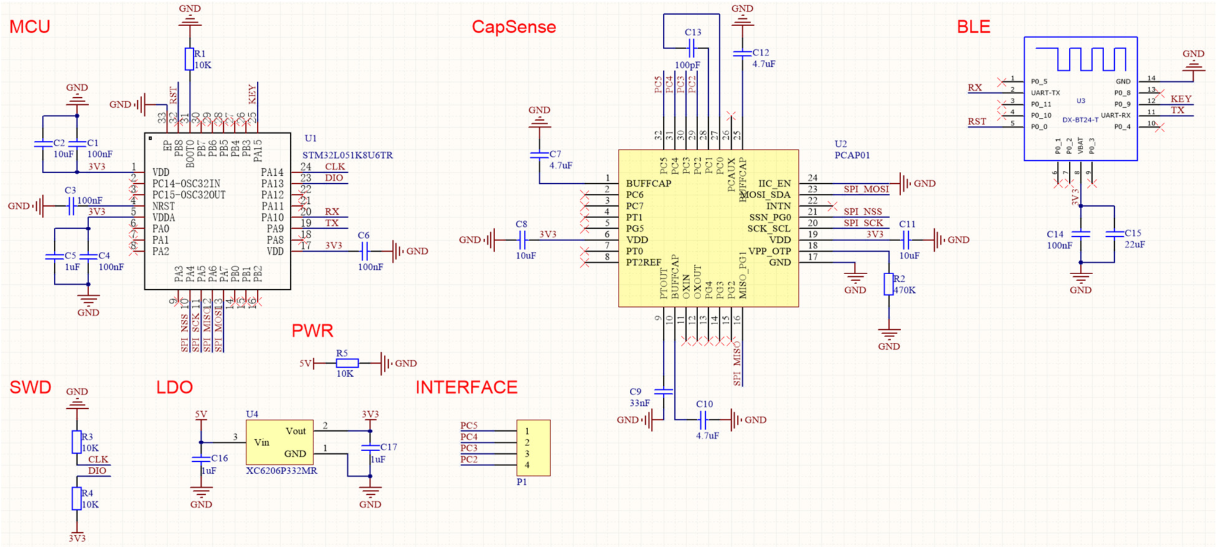

Fig. S2 Circuit schematic of the flexible circuit board.

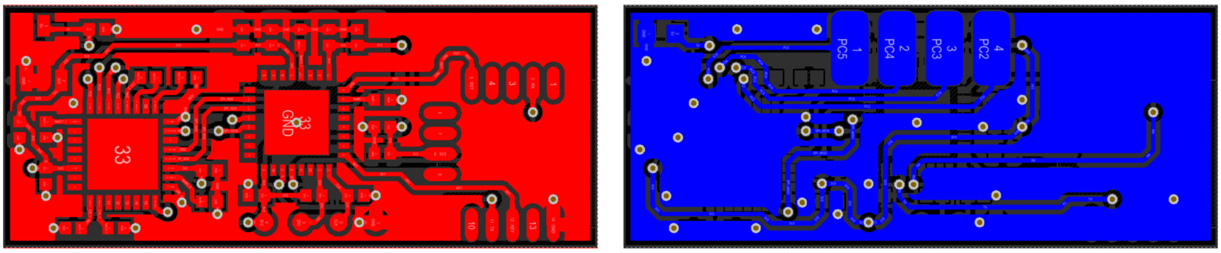

Fig. S3 PCB diagram of the flexible circuit board.

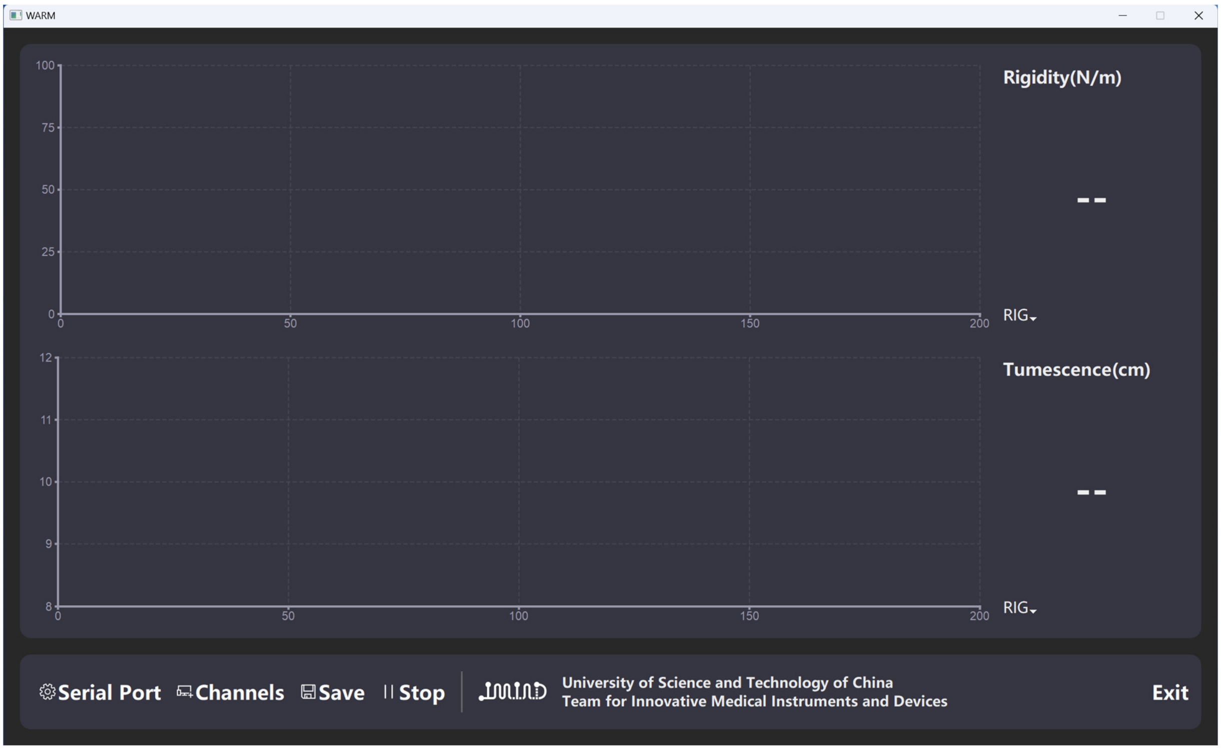

Fig. S4 Upper computer user interface of the WARM system.

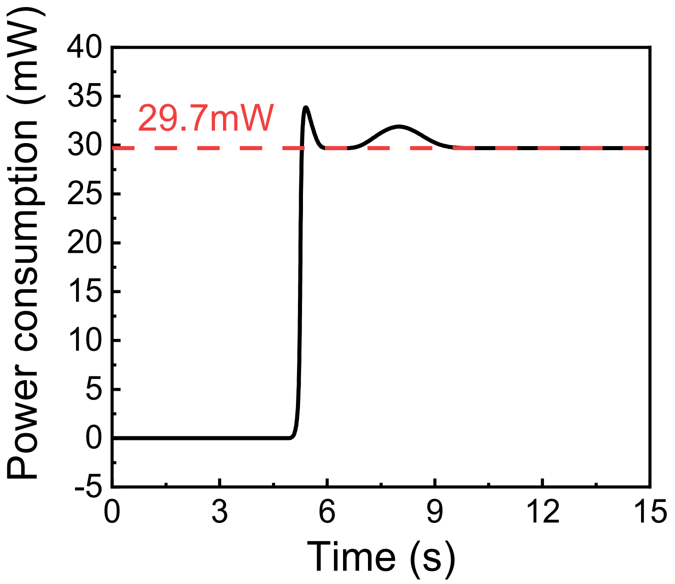

Fig. S5 Power consumption of the WARM system. The power consumption of the WARM system during normal operation is 29.7 mW.

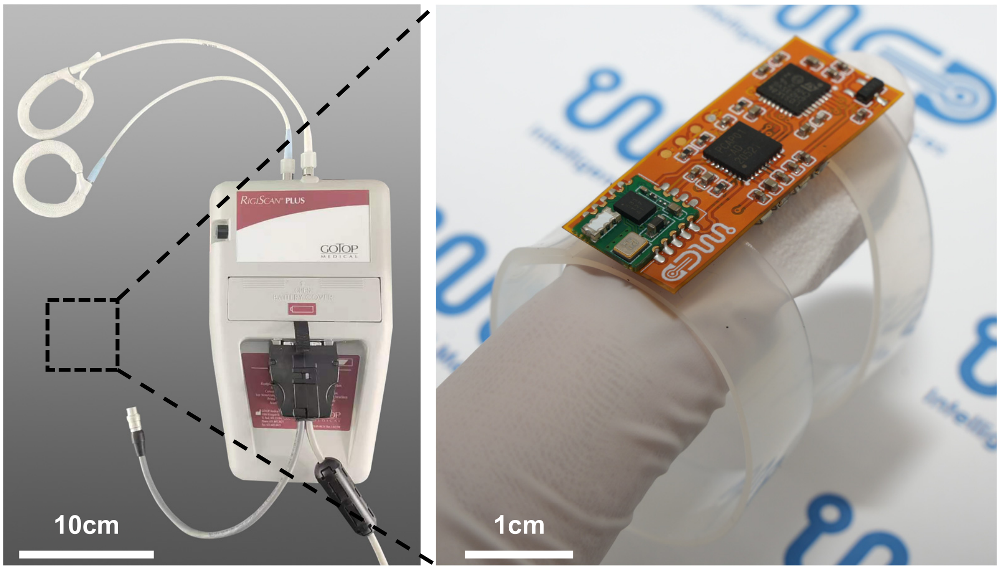

Fig. S6 Optical images of the RigiScan (left) with a scale bar of 10 cm, and the WARM system (right) with a scale bar of 1 cm. The RigiScan controller measures 13.34×21.60×5.08 cm, while the FPC of the WARM measures 1.3×3.2 cm.

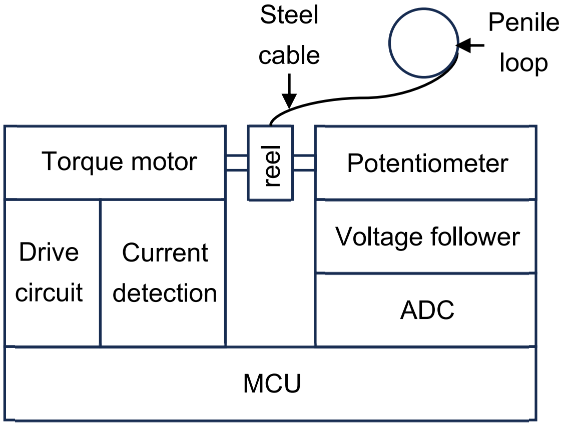

Fig. S7 Working principle of the RigiScan. The RigiScan drives the penile loop to shrink using a torque motor and measures the change in the penile loop's circumference with a potentiometer.

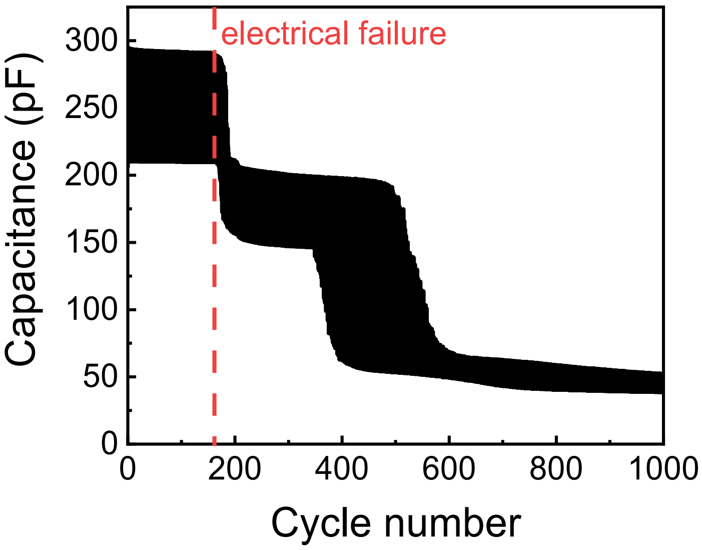

Fig. S8 Electrical failure of the sensor. After a certain number of stretching cycles, the sensor's capacitance decreases significantly and irreversibly.

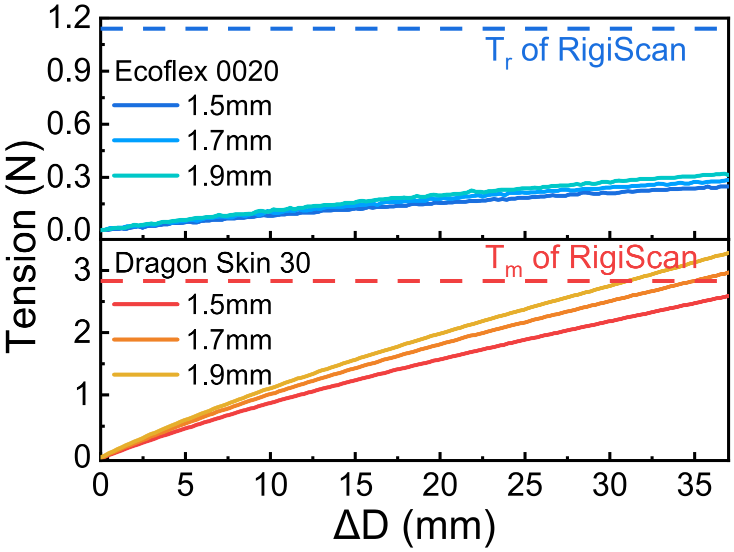

Fig. S9 Effect of thickness on the mechanical properties of silicone rubber samples made from Ecoflex 0020 and Dragon Skin 30. As the sample thickness increases, the maximum tensile load also increases.

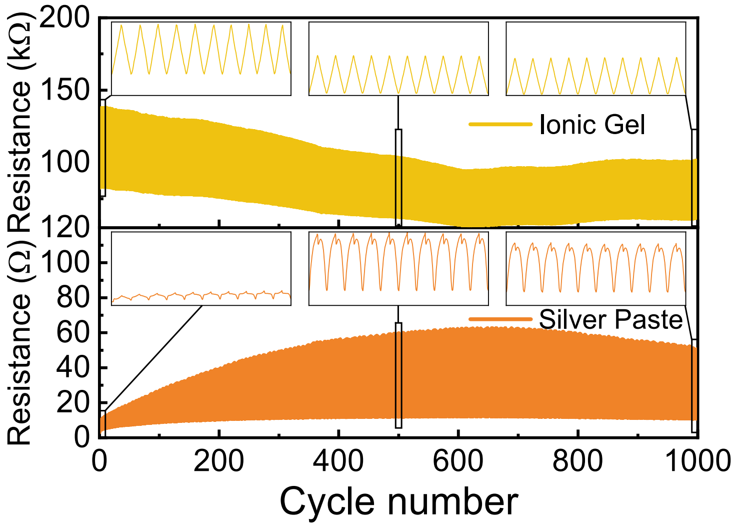

Fig. S10 Electrical performance of ionic gel and conductive silver paste over 1000 tensile cycles. Both the ionic gel and the conductive silver paste maintain good conductivity throughout 1000 tensile cycles.

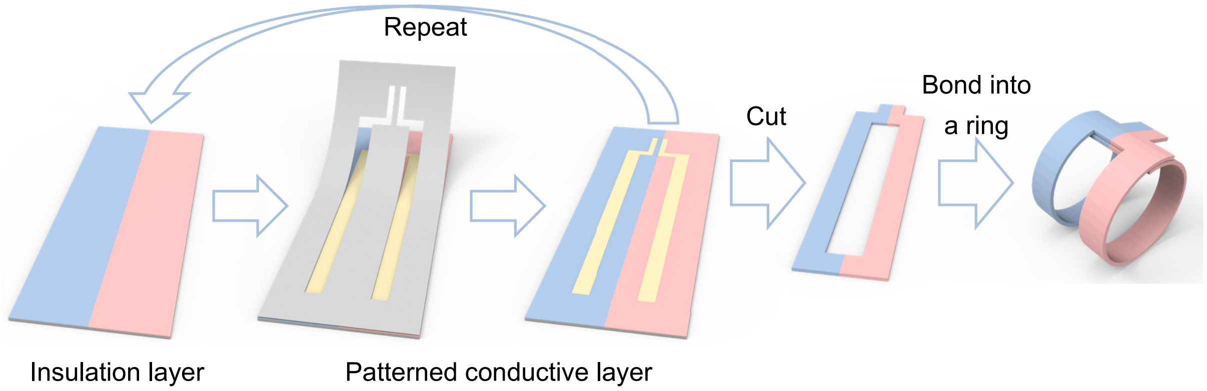

Fig. S11 Preparation process for the dual-ring sensor. A detailed description is available in the Methods section.

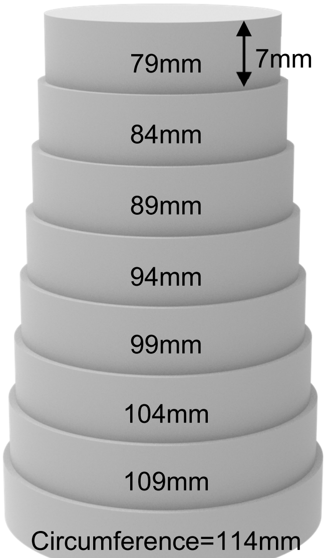

Fig. S12 3D-printed cylindrical model with varying circumferences for sensor calibration. The circumferences of the models are 79, 84, 89, 94, 99, 104, 109, and 114 mm, respectively.

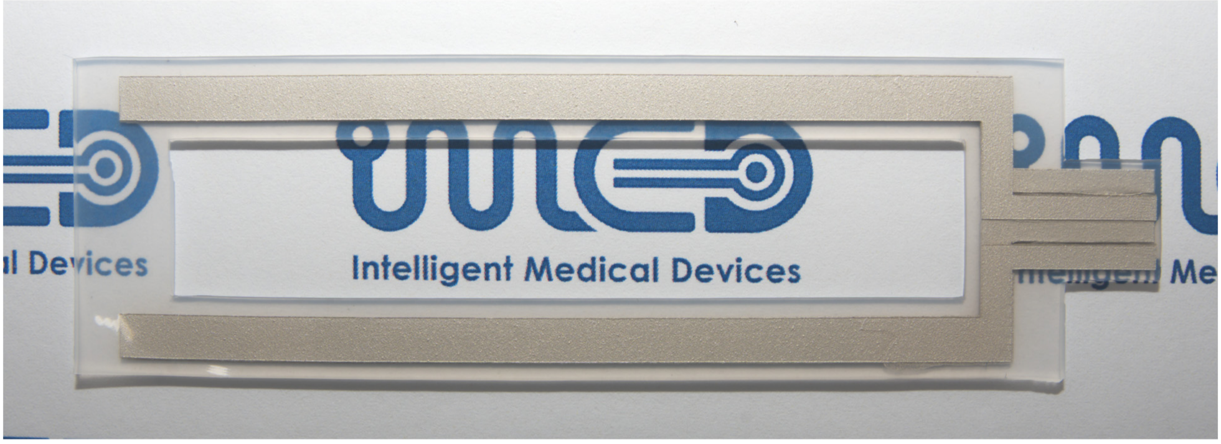

Fig. S13 Optical image of the dual-ring sensor using stretchable conductive silver paste for the conductive layer.

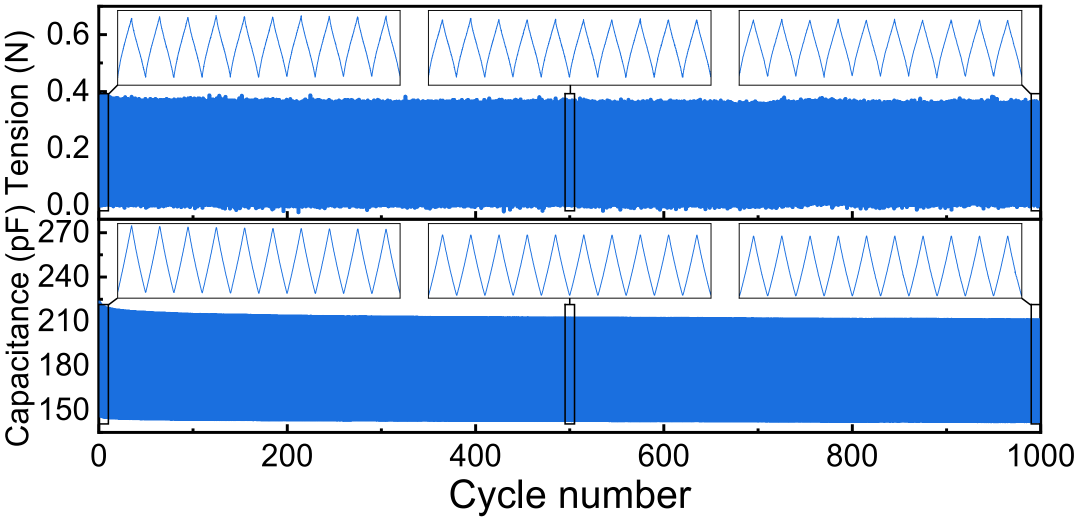

Fig. S14 Mechanical and electrical performance of the reference ring during 1000 tensile cycles.

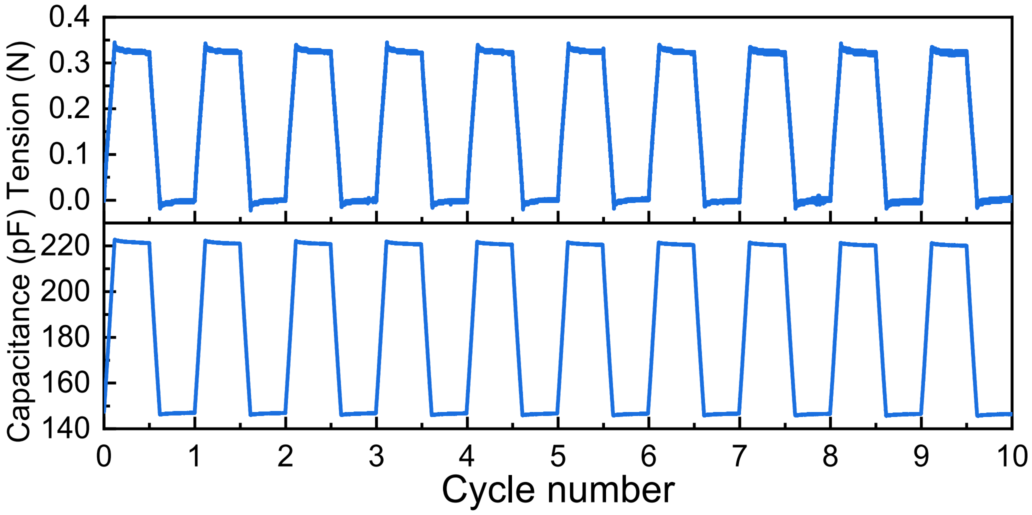

Fig. S15 Mechanical and electrical performance of the reference ring during the simulated phases of tumescence, sustenance, and detumescence.

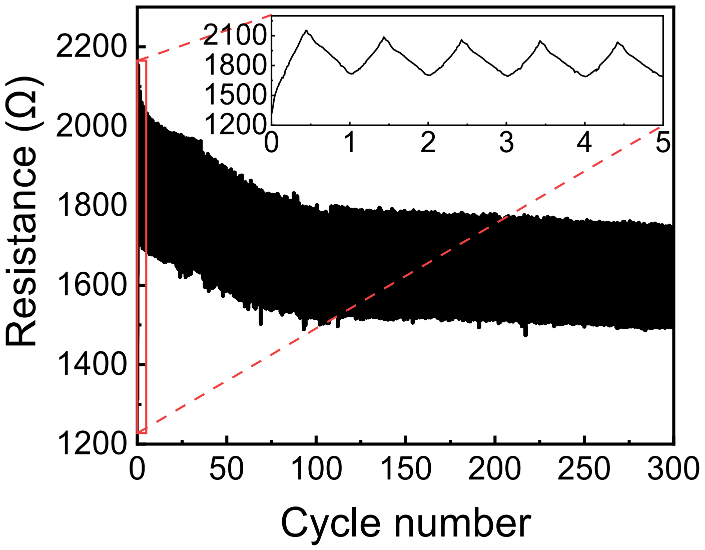

Fig. S16 Resistance response of commercial conductive rubber during tensile cycles. After 200 stretching cycles, the maximum resistance of the conductive rubber decreased by 19%. The initial resistance of the conductive rubber before the cycle started was 1312 Ω, and the resistance could not return to its initial value after recovery from tension.

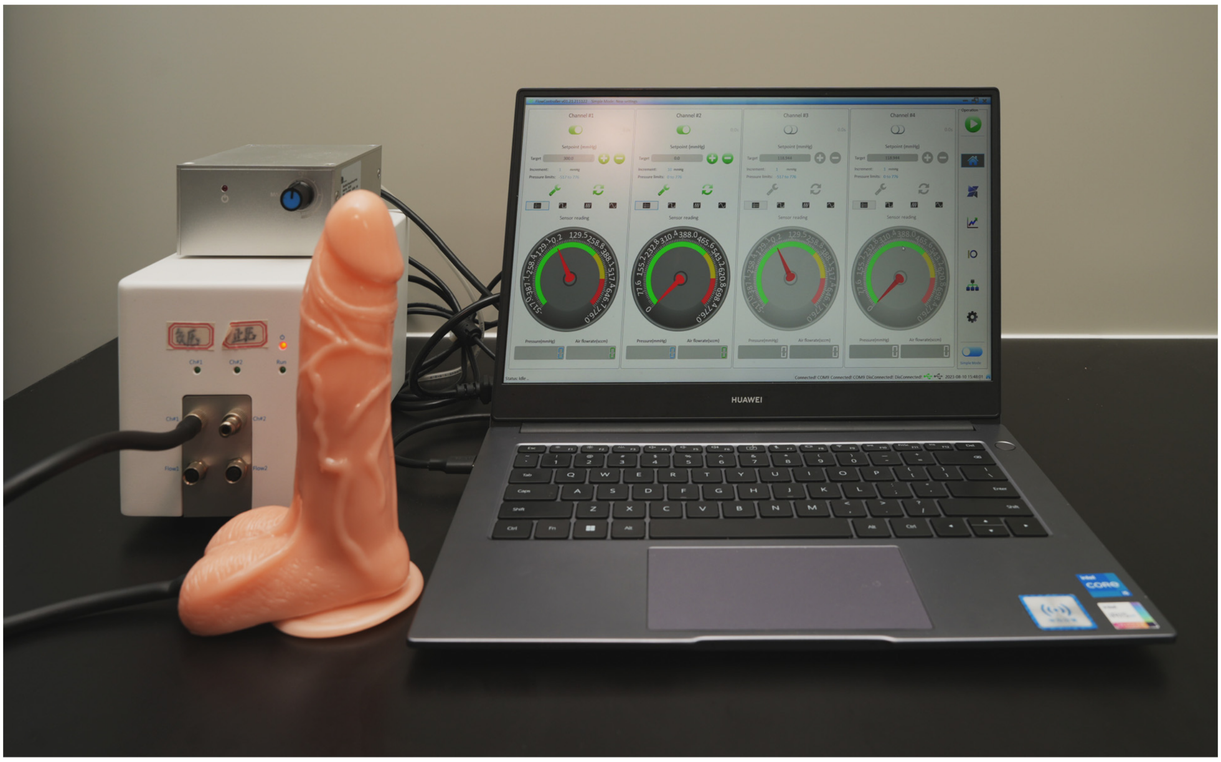

Fig. S17 Optical image of the penile tumescence simulator consisting of an expandable penile model, a flow controller for adjusting the model's rigidity and circumference, and a computer with the corresponding control software.

Elastomer	Elastic Moduli
Ecoflex	50-100kPa
Dragon Skin	150-600kPa
PDMS	0.5-3Mpa
TPU	3.6-88.8MPa
PI	2.3GPa
PET	2.5GPa

Table S1 Elastic moduli of common insulating elastomers.[3]

	Duration	Erection Duration	Erections	Initial Rig.	Erection Rig.	Initial Tum.	Erection Tum.
**WARM**	30min	12min	2	83.46N/m	182.88N/m	91.3mm	111.8mm
**RigiScan**	30min	11 min	2	-	71%	84mm	109mm

Table S2 AVSS test results recorded by the WARM system and the RigiScan.

References

1 Wang, C. T. Systematic review and comparative study on length and circumference of penis of adults at home and abroad. *Chinese Journal of Human Sexuality* **29**, 143-148 (2020).

2 Lee, P. A. Survey report: concept of penis size. *J Sex Marital Ther* **22**, 131-135 (1996).

3 Gillan, L., Hiltunen, J., Behfar, M. H. & Rönkä, K. Advances in design and manufacture of stretchable electronics. *Japanese Journal of Applied Physics* **61**, SE0804 (2022).
